# Influence of 50-Hz Electromagnetic Field on Anurian (Xenopus laevis) Metamorphosis

**DOI:** 10.1100/tsw.2004.177

**Published:** 2004-10-20

**Authors:** S. Grimaldi, A. Lisi, S. Reiti, V. Manni, M. Ledda, L. Giuliani

**Affiliations:** ^1^ Institute Neurobiology and Molecular Medicine, CNR, via del Fosso del Cavaliere, Rome, Italy; ^2^ ISPESL, via Urbana 167 00184. Rome, Italy

**Keywords:** low-frequency/low-intensity magnetic field, tadpoles, survival, embryogenesis

## Abstract

In this study, we show the effect of a 1-mT magnetic field AC at 50 Hz on *Xenopus* laevis tadpole populations. In the course of a 65-day exposure to the field, tadpole survival showed a small, but significant, decrease (p < 0.0004), together with a striking parallel 6-day shift in tadpole maturation frequency and a significant impairment of their metamorphosis. Particularly, metamorphosis was successful for 85% of individuals in the unirradiated tadpole population and for 45% of individuals in the irradiated tadpole population, respectively.

### INTRODUCTION

Life on Earth originated in the natural magnetic field (MF), but a large part of living matter became subjected to artificial electric, magnetic, and electromagnetic fields (EMF) only during the last century. For this reason, a question emerged regarding the adaptation of biological systems from exposure to these artificial fields: Would an organism, like an animal, maintain normal ontogenesis in an artificial MF characterized by an amplitude greater than that in which it evolved? The number of reports concerning this question has increased in recent years. The epidemiological aspect was debated with emphasis because the unhealthy effect of extremely low-frequency (ELF) MFs, generated by 50–60 Hz high-voltage power transmission lines, video display terminals, electric blankets or other home appliances, was observed[1,2]. The belief that the field intensity, in the case of these sources, of course, is rationally fixed below a risk threshold functioned as objection to the alarm. However, the available data were contradictory[3]. On one hand, low-energy pulsed MF benefits in nonunion bone fracture healing and in cell regeneration was reported[4]. On the other hand, the influence of a 50–60 Hz MF on nervous tissue induced preoccupation[2]. The EMF certainly showed some influence on cell proliferation[5,6] and on the role of $Ca^{2+}$ ions in the transduction of the influence itself[5,7,8,9,10,11]. Particularly, a 22-mT MF at 60 Hz modulated the $Ca^{2+}$ influx[9], while in turn the action of static MFs of the same order of intensity was correlated with programmed cell death via a $Ca^{2+}$ influx[12]. The MF dependence of the state of the cell membrane was investigated not only through electron microscopy[13], but also through cytofluorimetry[14]. The influence of 1–10 mT MFs at 50 Hz on the membrane conductivity and permeability was taken into consideration[15]. Lastly, a study was performed on the effect of very weak MFs ($10^{-6}$–$10^{-5}$ T) on chicken embryogenesis[16]. The results of this study were confirmed in several laboratories[17,18,19,20,21] and contradicted in others[22,23,24]. The present study has collected new information showing a heavy effect of a low-frequency/low-intensity MF on the embryogenesis of *Xenopus laevis*.

### MATERIALS AND METHODS

#### Eggs Fertilization

Three days before the time established for oviposition, an adult male *X. laevis* received an injection of 0.5 ml physiologic solution containing 300 units of gonadotropic hormones (Profasi HP, Serono, Rome) into the lymphatic dorsal sac. The same animal was treated again on the second and third days with an equal dose of gonadotropic hormones. In correspondence of the same third day, a *X. laevis* adult female received a dose of gonadotropic hormones into her lymphatic dorsal sac, which was the double of that received by the male (600 units in 10 ml of physiologic solution). The treatment had the purpose of guaranteeing mating and oviposition within the expected times. Stimulated male and female were introduced into an aquarium and maintained there overnight, in the dark, at a temperature of 21.0 ± 0.5°C. On the next day, fertilized eggs were laid. This day was considered as the initial time of tadpole population development.

#### Tadpole Growth

Soon after oviposition, *Xenopus* male and female were withdrawn from the aquarium and water was brought to a temperature of 26.0 ± 0.5°C, which was the optimal value for *X. laevis* embryo development. On the third day from oviposition (when the passage from embryo to larval stage occurred), the tadpole population was randomly subdivided into four groups, each of 200 individuals. These groups were transferred from the aquarium where the oviposition took place to four other fresh aquariums where the water temperature was also regulated at 26.0 ± 0.5°C. Two aquariums (irradiated tadpoles) were then exposed to a 50-Hz, 1-mT MF generated in a solenoid (SLD), while the other two aquariums (control tadpoles) were maintained outside the SLD itself. Experiments were performed in the course of the metamorphosis process, lasting approximately for 65 days. Four sets of experiments were performed.

#### Tadpole Maintenance

The tadpoles' daily diet was made up of an infusion of nettle, prepared as follows: 13 g of virus-free nettle powder, diluted in water, was put into a silken bag and boiled in a beaker for 40 min. After strong wringing in the boiled water, the silken bag was put into an oven at 100°C to determine the dry remains. The boiled water, holding an average of 10 g of nettle, was diluted into 600 ml of tap water and 300 ml of this nettle infusion was poured into an aquarium. The tadpole feeding required a periodic cleaning of the aquarium because the uneaten nettle particles, the organic waste of the tadpoles, and eventually the dead individuals could cause perturbation of the quality of the environment. To avoid these problems and to provide tadpoles with a satisfying oxygenation, the change of water was performed at 48-h intervals during the whole course of an experiment.

### Exposure System

Aquariums containing tadpoles were located in metacrylate amagnetic incubators (220 × 235 × 230 mm), assembled and patented by Eremenko et al.[25]. These incubators were regulated at the desired temperature by thermostated water baths. While the control incubators were maintained outside the artificial EMF, the experimental ones were introduced into a SLD generating sinusoidal EMF. This SLD, 960 mm long, was obtained by winding 620 turns of 1.5-mm diameter copper wire on a G10 fiberglass cylindric tube that was 360 mm in diameter, 1000 mm in length, and 8 mm in wall thickness[6].

A detailed scheme for the exposure of biological samples to the EMF was previously described[6]. In the present study, the SLD was directly supplied to the main power at 50 Hz, while a Variac was applied to give the desired EMF intensity. The MF AC in the SLD was monitored by using a Wandel Golthermann EFA-3 unit; where experimental incubators were positioned, the MF showed a more than 98% degree of homogeneity. An appropriate device guaranteed the constant temperature in SLD.

### Sampling and Calculation of Tadpole Survival

Tadpole survival was obtained by daily observation and count of individuals present in each aquarium.

## RESULTS

### Effect of MF on the Survival of Tadpoles

Survival value of *X. laevis* tadpoles was expressed in percentage. In both cases, outside or inside the SLD, this value went down up to 90% (Fig. 1). However, the rate of this survival-decreasing pattern was slightly faster for irradiated tadpoles when compared with nonexposed (control) tadpoles, especially during the first 10 days of irradiation. The same result was observed in four separate experiments; although extremely small, the average survival difference (2–4%) between the group of nonexposed individuals and that of the exposed ones showed to be fairly reproducible ($p < 0.0004$).

### Effect of MF on Tadpole Metamorphosis

Notwithstanding the significance of the *t* test, if the small difference of survival shown between both unirradiated and irradiated tadpoles was judged still *sub judice* for caution, the clear influence of the MF on the behavior of the tadpoles daily metamorphosis was of great interest (specifically, metamorphosis was completed when the tadpole tail was reabsorbed). When compared with the value of the unexposed tadpole, the frequency of the daily metamorphosis of the exposed ones was strikingly different. Comparing the shapes of the two curves in Fig. 1, it emerged that — in the course of about 60 days from the egg fertilization — two main phenomena were observable: (1) there was a continuous 5% decrease on daily metamorphosis average, in favor of the unirradiated (control) individuals; (2) there occurred a delay of 6 days affecting the optimal value in the daily metamorphosis of the irradiated tadpoles, when compared with unirradiated (control) tadpoles. In fact, while the timing for expression of an optimal daily metamorphosis in the unirradiated population coincided with the 46$^{th}$ day from egg fertilization, in the irradiated population this time coincided with the 52$^{nd}$ day. This is shown in Fig. 2, where the two curves increased in parallel up to the 35$^{th}$ day from egg fertilization; however, during the next 11 days (up to the 46$^{th}$ day), the daily metamorphosis essentially involved the unirradiated tadpoles only (in this case, daily metamorphosis ended relatively earlier, at the 53$^{rd}$ day); *vice versa*, during the next 10 days (up to the 52$^{nd}$ day), the daily metamorphosis essentially involved the exposed tadpoles only (in this case, daily metamorphosis continued alone up to the 59$^{th}$ day). Moreover, Fig. 3 shows that metamorphosis was

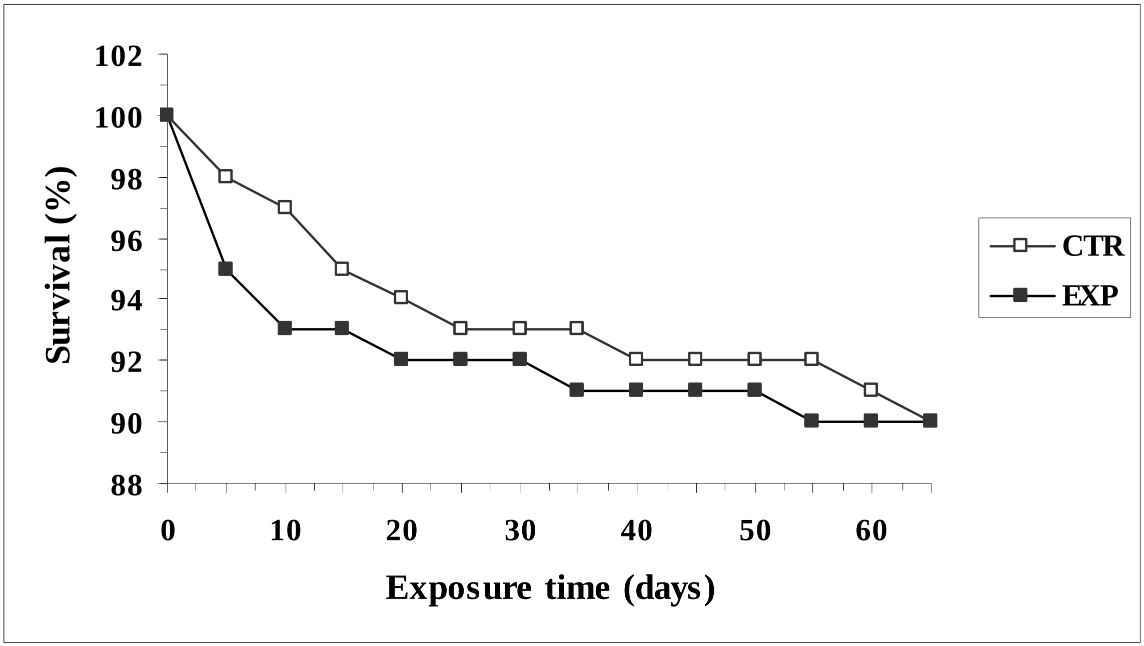

**FIGURE 1**. MF dependence of tadpole survival rate. (o) Control population made by unirradiated individuals maintained for 65 days outside the SLD; (•) experimental population made by individuals maintained for 65 days inside the SLD. The points represent the mean of four sets of experiments.

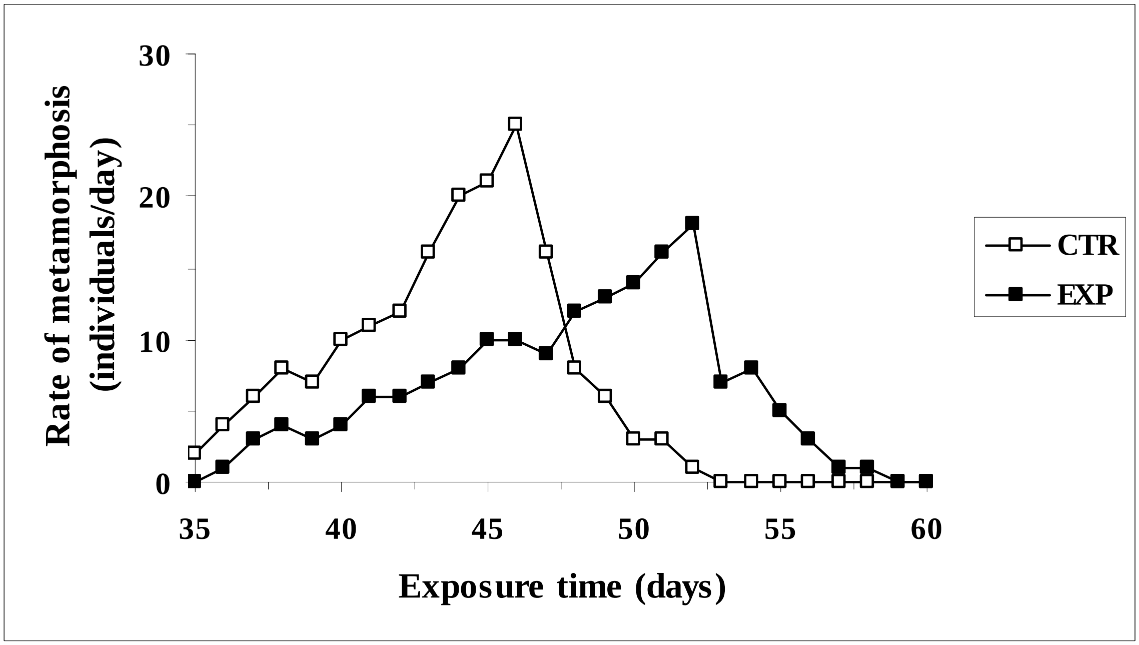

**FIGURE 2**. MF dependence of daily tadpole metamorphosis rate. As daily rate of metamorphosis, the number of individuals undergoing metamorphosis in 24 h was taken. (o) Control population made by unirradiated individuals maintained outside the SLD; (•) experimental population made by individuals maintained inside the SLD. The points represent the mean of four sets of experiments.

successfully completed for 85% of individuals in the unirradiated (control) tadpole population and for 45% of individuals in the exposed tadpole population.

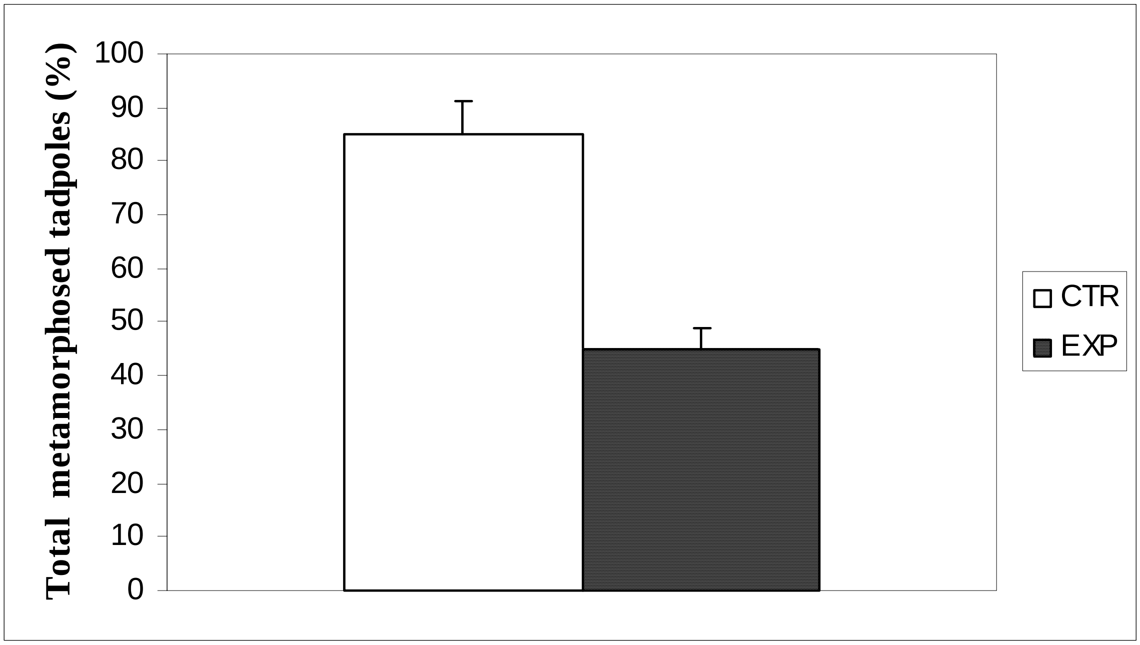

**FIGURE 3**. MF dependence of complete metamorphosis in the total tadpole population. The columns show the percentage of individuals that completed their metamorphosis during the experimental time. (1) Unirradiated (control) tadpoles; (2) irradiated tadpoles. The data represent the mean of four sets of experiments.

### DISCUSSION

This study involving populations of *X. laevis* tadpoles followed a similar work performed in our laboratory[26] on the biological effect of a continuous exposure of tadpoles EMF at 20 Gauss. In the conditions of a SLD irradiation generating a 1-mT EMF at 50 Hz, a small, but extended, reduction of tadpole survival during exposure to 1-mT EMF lasting for about 2 months still appeared, together with a reduction of the rate of tadpoles metamorphosis and an impairment in the total number of individuals able to complete their metamorphosis. The theoretical importance of these results would deal with the role that should be assigned to artificial MFs in relation to the evolution of living matter on the Earth and in relation to the problem of the biological adaptation to non-natural MFs[14]. On the other hand, the practical importance of these results would deal with the epidemiological problem that emerged when the technological advances added the MF "pollution" to its natural MF DC (45 μT). Anyway, by revealing an effect of a weak MF AC on a fast-developing population like *X. laevis* tadpoles, the results of the present work are consistent with those obtained in other laboratories[27,28]. Despite the problem of a possible separate biological role of the electrical component accompanying a MF AC having a value higher than that used here in the SLD (in water, for a 1-mT MF AC at 50 Hz, the electrical component was considered to be practically irrelevant), molecular and cellular mechanisms through which a weak ELF MF can slow the developmental rate and impair the metamorphosis at present can only be a speculation. From this viewpoint, this research was programmed to study the eventual MF dependence of the endocrine system functions, particularly of the anterior pituitary gland and of the thyroid, which in *X. laevis* are known to play a role in the development from the larval to the adult stages.

Moreover, these findings allow us to consider *X. laevis* populations as sensitive biological indicators of low-frequency MF effects.

### ACKNOWLEDGMENTS

The work was partially supported by a grant from the ISPESL-DIPIA, Rome.
